# Annotations

**Published:** 1887-12-03

**Authors:** 


### ANNOTATIONS.

**A Great Opportunity.**

THERE are those who argue that there should be some force in persistent operation for killing the superfluous population, and that it does not very much matter what it is, provided it be thoroughly effective. A murderous war or a sweeping and deadly pestilence are both well-known means to this end. But civilised man is becoming decidedly adverse to war, and sanitary man feels himself bound in honour to keep pestilence at bay ; so that those who see nothing but universal starvation in the unchecked increase of the people are beginning to feel more than a little dismayed. The Report to the Board of Trade, issued on Thursday, on the "Sweating System" at the East-end of London, by Mr. John Burnett, the labour correspondent to the Board, comes at a most opportune moment for the comfort of those far-seeing economists. No reflective man need for a moment doubt that in the scheme of Providence it is the usual and proper thing for the weak to go to the wall. Political economists have over and over again proved to a demonstration that the "law of supply and demand" must constantly operate in the regulation of human affairs to the practical exclusion of every other law, but especially of any law of a philanthropic kind ; and the establishment of the scientific principle of the "survival of the fittest" jumps so exactly with this conclusion that he who does not see the eternal wisdom and beauty of the arrangement stands self-confessed a mere uncultured lout and noodle. Those who desire to enjoy this best of all possible worlds, without the wretched little worries and annoyances arising from poor relations and miserable fellow-countrymen, are to be congratulated on the facts brought to light by Mr. Burnett's Report. It is undoubted, according to that encouraging document, that at the East-end of London, quite near to the wealthy West, there is going on a process of body and soul destruction rapid enough to satisfy the most voracious pauper-devourer in the world. The conditions of life therein detailed, including as they do long hours, small workrooms crowded to excess, unremitting toil, starvation wages, and a future absolutely destitute of hope—such conditions the common and inevitable lot of tens of thousands of men, women, and children, not only clear off all but the very strongest with appalling rapidity, but sow the seeds of dangerous diseases in such a fruitful soil that we may, at no distant date, expect the crop to flow over in all directions, to the probable decimation of the whole people, political economists included. The prospect we say is decidedly cheering, and the various classes of social philosophers who love to see their principles illustrated and carried out on a large scale should not only be supremely contented themselves, but should take advantage of the opportunity to give abundance of what the doctors call "clinical lectures" and "demonstrations" with the view to a rapid increase of converts to their cheerful and self-comforting faith. Such an occasion may not readily occur again in London, because a good and wholesome pestilence may at any time break out and entirely spoil the present highly instructive state of things.

**An Indelible Dishonour!**

IN the meantime those foolish philanthropists who are never content to let well alone will be much disturbed by Mr. Burnett's Report. It is reading of a kind to make a nervous man unhappy. A man or a woman foolish enough to admit the possession of a heart will read the plain statements of fact with mixed feelings of indignation, pity, and despair. "Sweating" is defined as a system under which sub-contractors undertake to do work in their own homes or small workshops, and employ others so to do it, making a profit for themselves by the difference between the contract prices and the wages they pay their assistants. These sub-contractors employ from one or two to forty or fifty persons, and appear to have formerly been more or less masters of their craft. The system is most largely developed in the tailoring trade ; but since the introduction of sewing machines and other modern appliances, it has undergone a complete change, so that now the great bulk of cheap tailoring is done by a class of men who need not necessarily be tailors at all, and who, as a matter of fact, very often know nothing about tailoring as an art. The demand for cheap clothing has tended constantly to lower the rate of wages, especially among the least skilled workers. Those unskilled workers include basters, machinists, pressers, fellers, buttonhole workers, and general workers. All these, with the skilled cutter, combine to produce the cheap clothing which fills the market. So that now we have between the consumer and the producer, the master tailor, the contractor, the sub-contractor, and, in some cases, several other middlemen, all requiring a profit more or less large. The learning of any of the different branches of tailor-work just enumerated except cutting is very easy, and vast numbers of persons have rushed into the trade as the easiest available refuge from starvation. Add to this the fact that in recent years there has been an enormous influx of pauper foreigners—chiefly German and Russian Jews—and it will be easily seen that, the work being more or less of a fixed quantity, and the workers being practically innumerable, there is a condition of the market which leaves labour utterly powerless and capital master of the field. In the tailoring trade alone there are said to be nearly twenty thousand pauper Jews at the East-end of London, besides other foreigners and native workers. No wonder that the miserable work-rooms are described in the Report as "sweating dens." Mr. Burnett says: "In rooms not more than nine or ten feet square, heated by a coke fire for the pressers' irons, and lighted with flaring gas jets, may be seen six, eight, ten, or twelve persons at work. The places are garrets, back rooms, cellars, etc., in the worst kind of East-end tenements, and it is impossible for any inspector to find them all out, especially as he is regarded as the common enemy. The hours of work are from twelve to fourteen hours a day for the women, and sixteen or even eighteen for the men." That such a state of things should exist at this period, and in a wealthy country like ours, is an indelible disgrace to every man and woman of wealth and leisure in the nation.

**Are we a Capable People?**

THE man or woman who seeks to know the condition of the poor for the mere purpose of making sensational newspaper "copy" deserves everybody's contempt, and to be kicked out of every workshop or casual ward into which he may put his nose. But the condition of the poor ought to be made known, and can usually be best made known by the newspaper writer, who yet may himself be totally unable to do anything effective towards the amelioration of their condition. We feel a kind of shame in drawing attention for the thousandth time to facts which everybody knows, and which have been forced upon the public *ad nauseam*. Nevertheless it seems imperative that some man or woman should take up the cause of the oppressed and helpless workers, as Howard took up the cause of the miserable prisoners, and Wilberforce, Clarkson, and others that of the slaves. There are far more men and women of wealth and leisure in England at present than there have been at any previous time. In London there is more wealth now, and more people who spend their days in idleness, than have ever been known at any other time or place in the world's history ; and yet the question of the "residuum" is almost untouched ! Can it be that wealth and leisure are destructive to self-denial and force of character? It would seem so. The subject presents this appearance—that of all the thousands, nay, tens of thousands, of rich and leisured persons in London, there is not, and has not been for years, a single one who can make himself a great and effective leader in the cause of the poor. We are not forgetting the late Lord Shaftesbury and others ; but the time demands statesman-like administrators, and not merely philanthropists. Here is a cause great beyond comparison. He who should convert the residuum into self-supporting, self-respecting citizens, would be worthy of greater honour than poet, soldier, or statesman ever received. Where is the capable man ?

